# Reduction of Artefacts in JPEG-XR Compressed Images

**DOI:** 10.3390/s19051214

**Published:** 2019-03-09

**Authors:** Kai-Lung Hua, Ho Thi Trang, Kathiravan Srinivasan, Yung-Yao Chen, Chun-Hao Chen, Vishal Sharma, Albert Y. Zomaya

**Affiliations:** 1 Department of Computer Science and Information Engineering, National Taiwan University of Science and Technology, Taipei 10607, Taiwan; hua@mail.ntust.edu.tw (K.-L.H.); d10515804@mail.ntust.edu.tw (H.T.T.); m10015902@mail.ntust.edu.tw (C.-H.C.); 2 Center for Cyber-physical System Innovation, National Taiwan University of Science and Technology, Taipei 10607, Taiwan; 3 School of Information Technology and Engineering, Vellore Institute of Technology, Vellore 632 014, India; 4 Graduate Institute of Automation Technology, National Taipei University of Technology, Taipei 10607, Taiwan; yungyaochen@mail.ntut.edu.tw; 5 Department of Information Security Engineering, Soonchunhyang University, Asan-si 31538, Korea; vishal_sharma2012@hotmail.com; 6 School of Computer Science, The University of Sydney, Sydney, NSW 2006, Australia; albert.zomaya@sydney.edu.au

**Keywords:** JPEG XR, HD photo, Windows Media Photo, chequerboard artefacts, border artefacts, corner block artefacts

## Abstract

The JPEG-XR encoding process utilizes two types of transform operations: Photo Overlap Transform (POT) and Photo Core Transform (PCT). Using the Device Porting Kit (DPK) provided by Microsoft, we performed encoding and decoding processes on JPEG XR images. It was discovered that when the quantization parameter is >1-lossy compression conditions, the resulting image displays chequerboard block artefacts, border artefacts and corner artefacts. These artefacts are due to the nonlinearity of transforms used by JPEG-XR. Typically, it is not so visible; however, it can cause problems while copying and scanning applications, as it shows nonlinear transforms when the source and the target of the image have different configurations. Hence, it is important for document image processing pipelines to take such artefacts into account. Additionally, these artefacts are most problematic for high-quality settings and appear more visible at high compression ratios. In this paper, we analyse the cause of the above artefacts. It was found that the main problem lies in the step of POT and quantization. To solve this problem, the use of a “uniform matrix” is proposed. After POT (encoding) and before inverse POT (decoding), an extra step is added to multiply this uniform matrix. Results suggest that it is an easy and effective way to decrease chequerboard, border and corner artefacts, thereby improving the image quality of lossy encoding JPEG XR than the original DPK program with no increased calculation complexity or file size.

## 1. Introduction

In a smart city environment, a colossal quantity of image data is generated from traffic control systems, intruder detection and surveillance systems and from other intelligent sensing gadgets and devices. Therefore, sending the data across the network might consume very high network traffic; hence, these images are compressed using suitable compression approaches such as Joint Photographic Experts Group (JPEG), JPEG Extended Range (JPEG-XR) and so on [1,2,3]. JPEG-XR was initially presented under the name Windows Media Photo, and subsequently, it was retitled as High Definition (HD) Photo. Further, in 2006, Microsoft proposed it as a compression method and file format for images. After that, in 2009, it was announced as the International Organization for Standardization (ISO)/International Electrotechnical Commission (IEC) and International Telecommunication Union (ITU) worldwide standard by the Joint Photographic Experts Group (JPEG) [4,5,6]. JPEG-XR has better compression efficiency than JPEG [7,8]. Its advantages include lossless compression, transparency, and support for multiple image formats. Moreover, at low bit rates, JPEG images usually suffer from blocking artefacts [9,10,11]. In comparison with JPEG 2000, JPEG-XR is less complicated; however, the bit rate for a given Peak Signal-to-Noise Ratio (PSNR) is comparable to the bit rate of JPEG-2000 and could be as much as 50 percent lower than the bit rate of JPEG [12]. Furthermore, the compression algorithm of JPEG-XR is conceptually identical to that of JPEG [13]. In the JPEG-XR process, an image is segregated into macroblocks of fixed size. These macroblocks in the spatial domain are then transformed into the frequency domain; the frequency components are then quantized and the entropy encoded [14].

The reversible integer transform is amongst the most significant features of the JPEG-XR compression standard. Further, this feature relies on two transform operators, namely the Photo Core Transform (PCT) [15] and the Photo Overlap Transform (POT). Moreover, the process involved in PCT is identical to the Discrete Cosine Transform (DCT) methodology, where the image is transformed into corresponding frequency groups [16,17]. The output image from the JPEG-XR process has blocking artefacts, which is similar to the discrete cosine transform approach. In order to overcome this limitation, the photo overlap transform is devised in such a way that it utilizes the correlation between the boundaries of the blocks for reducing or removing the blocking artefacts [18]. Moreover, the operators are devised by using estimates of rotation operations using linear shears. As a result, the chequerboard artefacts are produced due to these estimates. Furthermore, given an uncompressed image with constant-intensity areas, the output of the JPEG-XR process might result in intensity variations similar to chequerboard artefacts. Nevertheless, these artefacts cannot be prominently observed through human vision. However, it might be a severe challenge in image document processing pipelines. Assume, for instance, an auto-cropping technique can be used by a scanner for removing the regions beyond a predefined threshold value [19,20]. Auto-cropping might not happen when there is the presence of the chequerboard artefact in the output image since the intensity values are below the predefined threshold. Furthermore, in the printing process of an image, the nonlinearities are another reason for augmenting the chequerboard artefacts. Similar to these discussions, some of the applications of the inverse problems, such as joint reconstruction of kinetic parametric images [21], reconstruction of volumetric myocardial [22] and noninvasive computational imaging [23], can be followed to further understand the advantages of inverse solutions.

Furthermore, in JPEG-XR, POT and PCT are implemented by the lifting structure [24,25]. This allows transform operations to be broken down into simple integer additions and subtractions (shears). This effectively lowers the computational complexity and resources consumed during the encoding and decoding process, which is especially advantageous on mobile or embedded devices where power is often limited. This implementation also offers reversibility so that lossless encoding can be performed. Inverse transforms can be easily implemented by sign reversal and the reciprocal.

Microsoft began offering the download of the DPK (Device Porting Kit) [26] at the end of 2006 for research and development purposes. While encoding images using this tool, we discovered that when the quantization parameter is >1-lossy compression conditions, the resulting image showed chequerboard block artefacts, border artefacts and corner artefacts. The artefacts are less obvious when the compression ratio is low (Quantization Parameter (QP) < 41). However, at a medium to high compression ratio (QP ≥ 41), they become more apparent, especially on pure colour (monochrome) images where the artefacts can be observed directly by the naked eye. While the artefacts do not significantly lower the image quality, they could cause annoyance when processing images in special situations. These could include post-processing of scans or reproduced photos, identification of characteristics or defects in computer vision, medical analysis and geographic analysis.

In Section 2 of this paper, we will analyse chequerboard block artefacts, border artefacts and corner artefacts respectively. Through implementation and observation, we will speculate about possible causes and prove them mathematically. In Section 3, we will design a separate “uniform matrix” for each of the three artefacts in order to improve encoding quality. In Section 4, we will implement the aforementioned uniform matrices and compare the results with Microsoft’s DPK.

It will become apparent that our method effectively removes chequerboard, border and corner artefacts and provides better image quality and compression ratio.

## 2. Chequerboard, Border and Corner Block Artefacts

**Figure 1:**
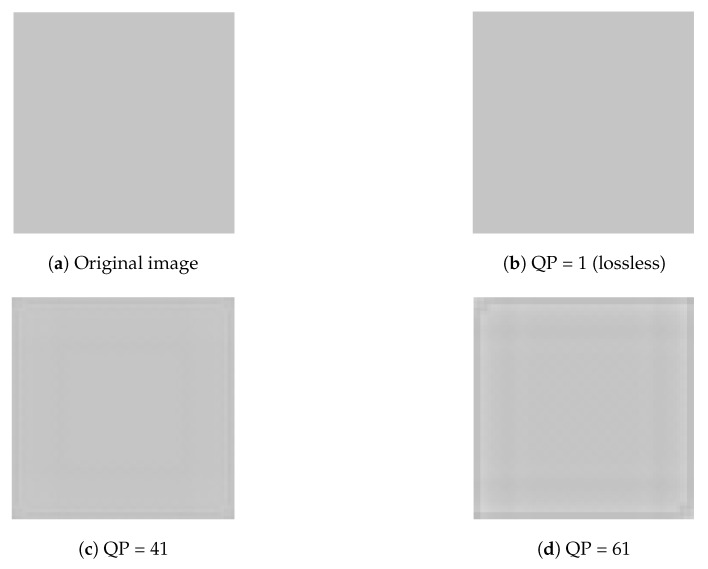
Part 1. Chequerboard block artefacts. QP, Quantization Parameter.

**Figure 2:**
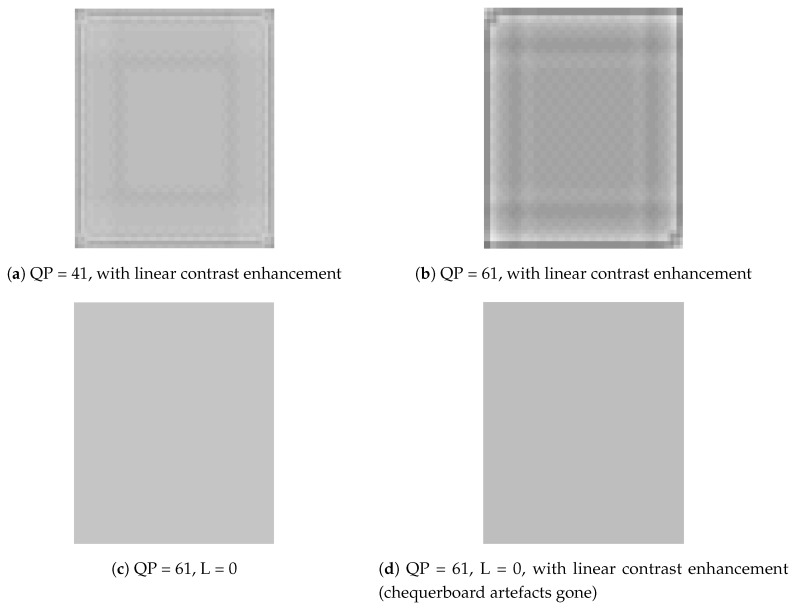
Part 2. Chequerboard block.

We used the DPK provided by Microsoft to encode and decode images. We discovered that chequerboard, border and corner artefacts occur when the QP >1. Figure 1a depicts the original image before transformation. It is a 64 × 64 monochrome bitmap (BMP). Figure 1b portrays a lossless transform using QP = 1. It is identical to the original image. Figure 1c shows a lossy transform using QP = 41. The difference between it and the original is not very apparent. Figure 1d illustrates a lossy transform using QP = 61. The chequerboard, border and corner artefacts are more obvious than in Figure 1c. Figure 2a,b shows the results of linear contrast enhancement ([1] $f_{ENC}\left(p\left[i\right]\right)\;=\;min\left(p\right)\;+\;{\left(p\left[i\right]\;-\;min\left(p\right)\right)}^{*}R$, where *p* = pixel array of image, i = array-index in p, R = 6 (expand ratio, fixed in this paper)) in Figure 1c,d. Chequerboard, border and corner artefacts are very noticeable. Note that if we set the overlap parameter L = 0, the artefacts disappear even without transformation with POT, as shown in Figure 2c,d. We can infer from the above discussion that the two causes of the artefacts are:

1. lossy quantization (QP > 1)
2. the use of POT (L > 0)

Hereinafter, we will analyse each of the two causes.

### 2.1. Lossy Quantization and Irreversibility

**Figure 3:**
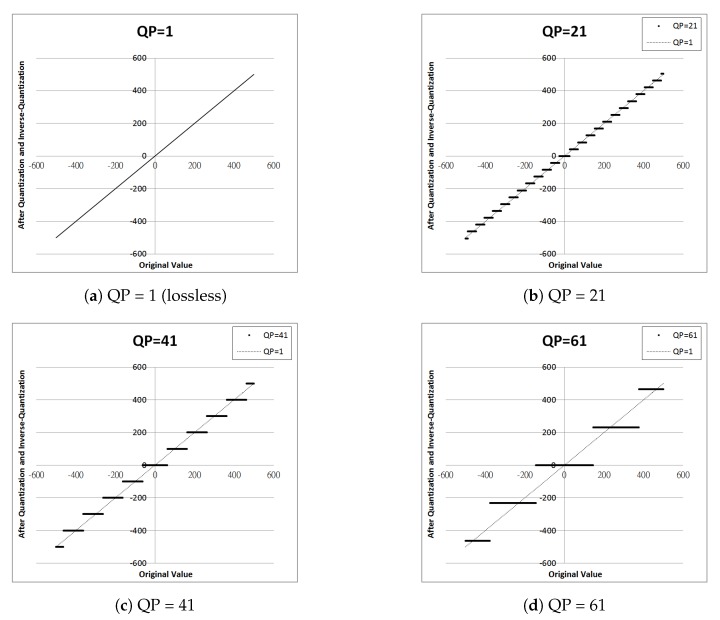
Lossless (QP = 1) and lossy (QP > 1) quantization.

Quantization of DPK is lossless when the quantization parameter QP = 1, as shown in Figure 3a. When QP > 1, the result is similar to uniform quantization, as shown in Figure 3b–d. The solid lines are the quantization results of QP = 21, QP = 41, and QP = 61, respectively. It can be observed that as QP increases, the interval (step) becomes wider.

**Figure 4:**
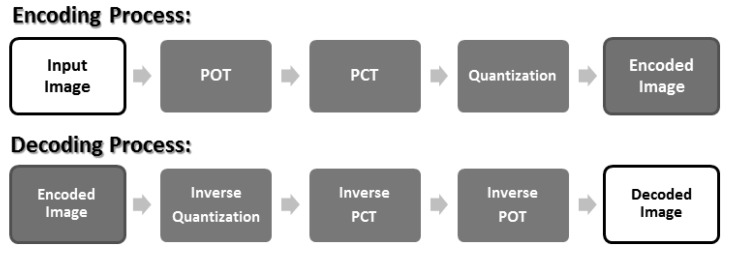
JPEG-XR encoding/decoding process. POT, Photo Overlap Transform; PCT, Photo Core Transform.

**Figure 5:**
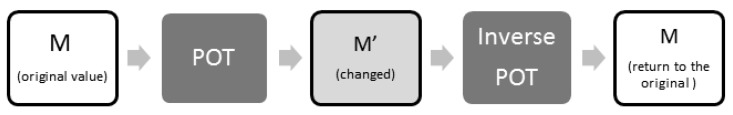
The “reversibility” of POT (and PCT).

The process of JPEG-XR encoding is as shown in Figure 4. From [18], we know that the POT and PCT implemented by the lifting structure are “reversible”. This means that after a value has been transformed by POT or PCT, we can apply the corresponding inverse transforms to return it to its original value, as shown in Figure 5.

**Figure 6:**
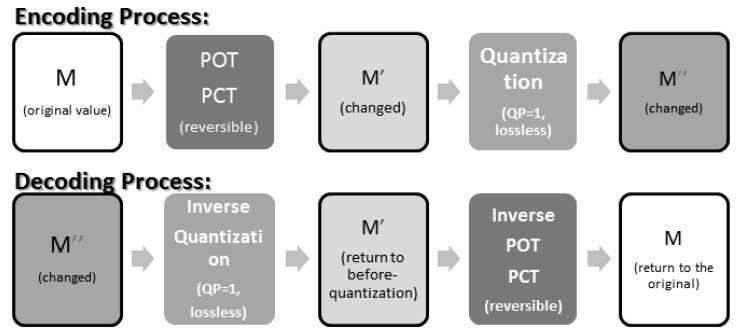
The “reversibility” when QP = 1.

When the quantization parameter QP = 1, the quantization is both lossless and reversible. With QP = 1 and the reversibility of POT and PCT, the encoding/decoding process of JPEG-XR becomes completely lossless. This way, no chequerboard, or border, artefacts will be produced, as shown in Figure 6.

**Figure 7:**
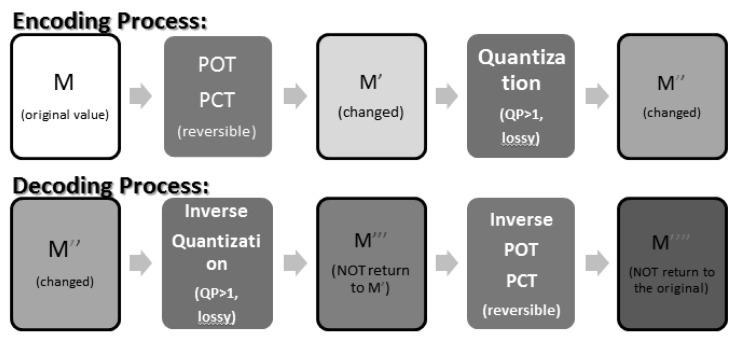
The “irreversibility” when QP > 1.

On the other hand, when QP > 1, or when quantization is lossy, the values may be offset or distorted. The inverse PCT, POT step is then irreversible and may cause chequerboard, border and corner artefacts, as shown in Figure 7.

### 2.2. POT

**Figure 8:**
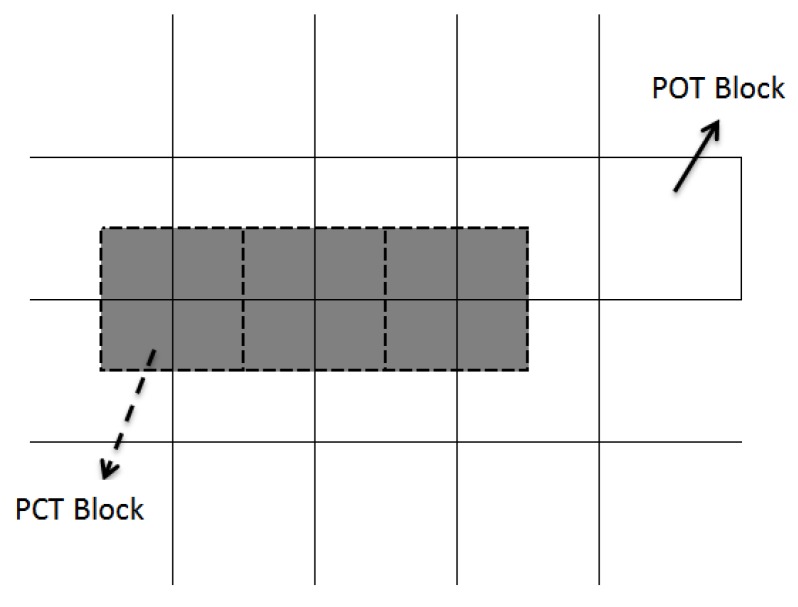
POT and PCT blocks interleave.

The purpose of JPEG-XR’s POT is to decrease block artefacts caused by PCT. As such, its application area must interleave with that of PCT [18], as shown in Figure 8.

**Figure 9:**
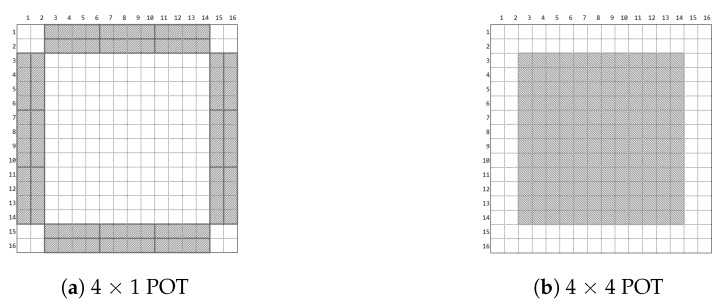
Two different types of POT application areas represented in grey.

**Figure 10:**
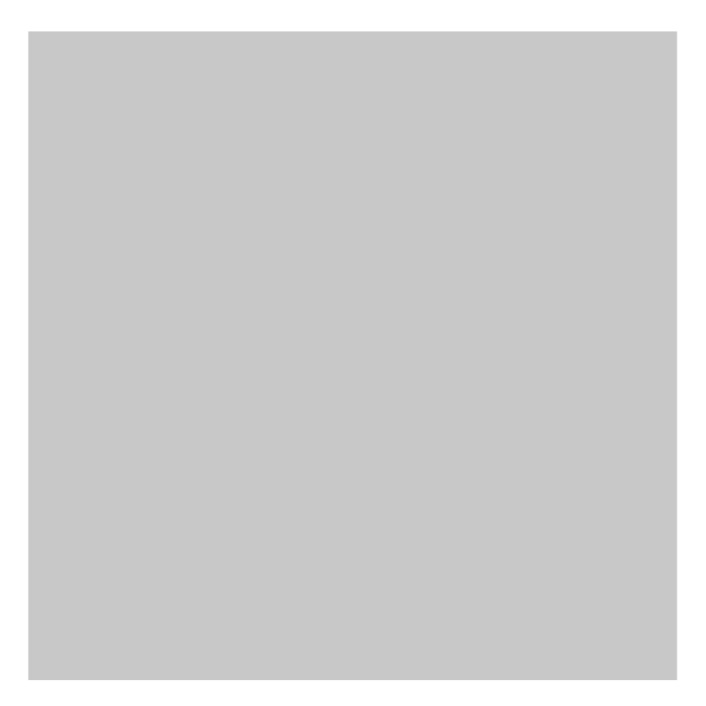
Original image, 16 × 16 grey BMP.

**Figure 11:**
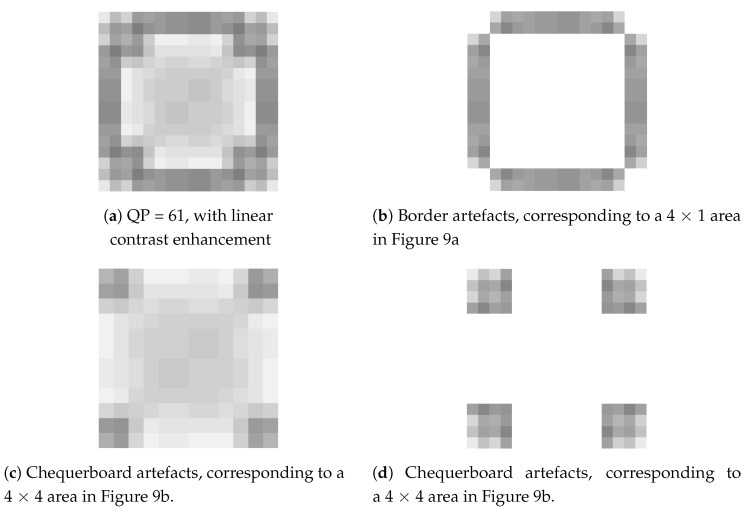
Chequerboard and border artefacts correspond to the two POT application areas, while the corner artefacts correspond to the four missing corners where POT was not applied.

Since POT must interleave with PCT when undergoing a POT, the image must be divided into two regions. To each of the two regions would be applied a different transform as shown in Figure 9a, depicting the border with 4 × 1 POT, while Figure 9b portrays the 4 × 4 POT inside. The location of the chequerboard and border artefacts happens to be where the 4 × 4 POT and 4 × 1 were applied. Additionally, the corner artefacts are located at the 42 × 2 corners where POT was not applied, as shown in Figure 10 and Figure 11.

Through research, we found the causes of the chequerboard, border and corner artefacts:

1. The 4 × 4 POT causes chequerboard artefacts
2. The 4 × 1 POT causes border artefacts
3. The four corners without POT application causes corner artefacts

Next, we will describe each of the three causes in detail.

### 2.3. The 4 × 4 POT: Chequerboard Block Artefacts

Based on [24], we know that the operations of 4 × 4 POT are separated into four stages, which by using the 16 points, a, b, c, d, e, f, g, h, i, j, k, l, m, n, o and p, are given as:

1. Hadamard transform stage:
  (a) $T_{HH}\left(a,d,m,p\right)$
  (b) $T_{HH}\left(b,c,n,o\right)$
  (c) $T_{HH}\left(e,h,i,l\right)$
  (d) $T_{HH}\left(f,g,j,k\right)$
2. Scaling stage:
  (a) $T_{S}\left(a,p\right)$
  (b) $T_{S}\left(b,l\right)$
  (c) $T_{S}\left(e,o\right)$
  (d) $T_{S}\left(f,k\right)$
3. Rotation stage:
  (a) $T_{R}\left(n,m\right)$
  (b) $T_{R}\left(j,i\right)$
  (c) $T_{R}\left(h,d\right)$
  (d) $T_{R}\left(g,c\right)$
  (e) $T_{RR}\left(k,l,o,p\right)$
4. Hadamard transform stage:
  (a) $T_{HH}\left(a,d,m,p\right)$
  (b) $T_{HH}\left(b,c,n,o\right)$
  (c) $T_{HH}\left(e,h,i,l\right)$
  (d) $T_{HH}\left(f,g,j,k\right)$

**Figure 12:**
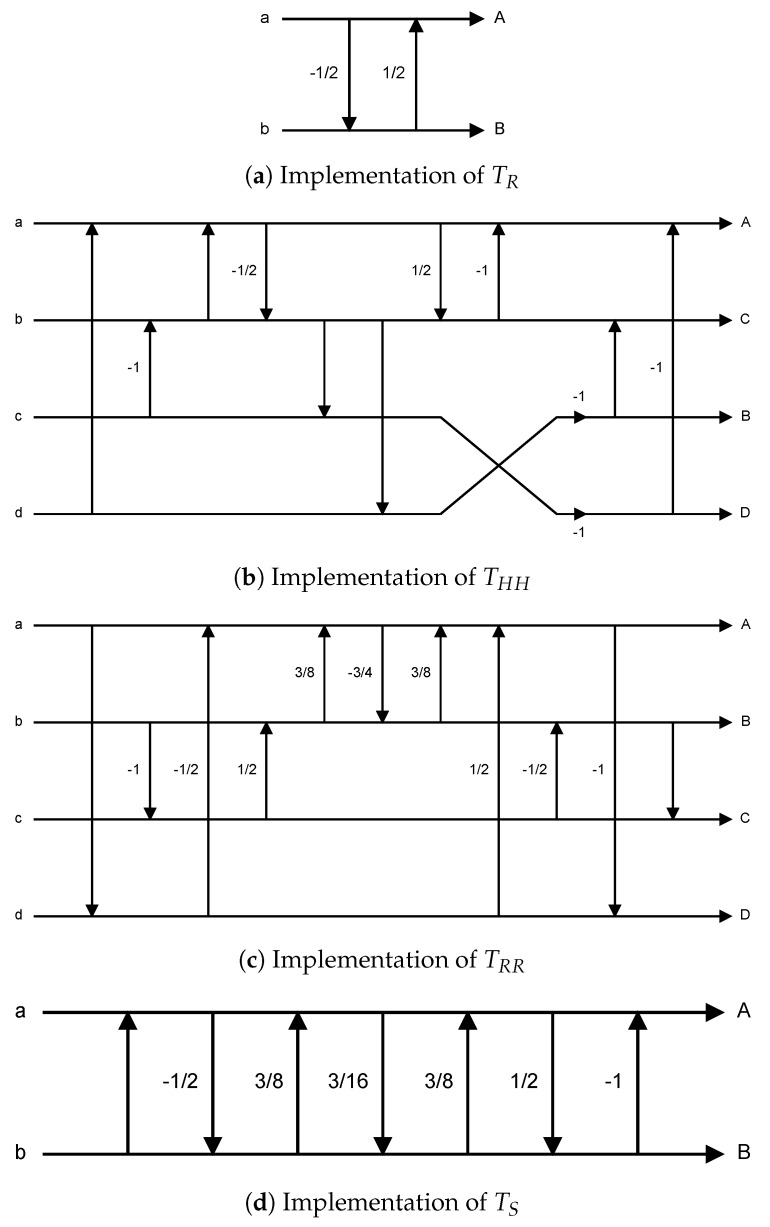
Implementations of JPEG XR POT rotation operators in Microsoft DPK.

Each of the above stages uses four rotational operators: $T_{R}$, $T_{HH}$, $T_{RR}$ and $T_{S}$. Figure 12a–d shows the implementations of $T_{R}$, $T_{HH}$, $T_{RR}$ and $T_{S}$ in DPK [26], respectively.

**Figure 13:**
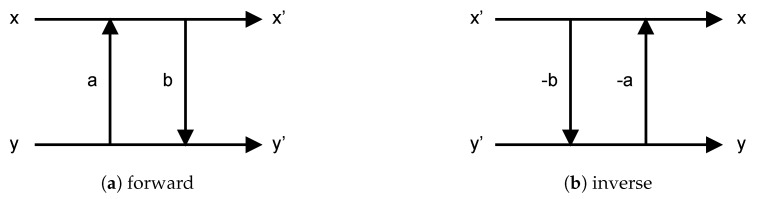
Lifting structure.

From [18], we know that the four rotational operators listed in Figure 12 are implemented by the lifting structure [24,25,27]. Its basic structure is as shown in Figure 13. The advantage of using the lifting structure is that the transform operations can be separated into simple addition, subtraction and multiplication (e.g., Figure 13a can be expanded into Equation (1)). Moreover, inverse transform operations can be achieved by simply reversing the operation order, and switching the pluses and minuses (e.g., Equation (2) is the result of expanding Figure 13b).

(1) $$\begin{array}{c} x^{\prime }=x+a^{*}y\\ y^{\prime }=y+b^{*}x^{\prime } \end{array}$$

(2) $$\begin{array}{c} y=y^{\prime }-b^{*}x^{\prime }\\ x=x^{\prime }-a^{*}y \end{array}$$

Using the method of Equation (1) and Figure 13a, we substitute from Figure 12a–d for the four POT rotational operators—$T_{R}$, $T_{HH}$, $T_{RR}$, $T_{S}$—and expand each. The results are shown in Equations (3)–(6).

(3) $$T_{R}\left(a,b\right)=\left[\begin{array}{cc} 3/4 & 1/2\\ -1/2 & 1 \end{array}\right]•\left[\begin{array}{c} a\\ b \end{array}\right]$$

(4) $$T_{HH}\left(a,b,c,d\right)=1/2\left[\begin{array}{cccc} 1 & 1 & 1 & 1\\ 1 & -1 & 1 & -1\\ 1 & 1 & -1 & -1\\ 1 & -1 & -1 & 1 \end{array}\right]•\left[\begin{array}{c} a\\ b\\ c\\ d \end{array}\right]$$

(5) $$T_{RR}\left(a,b,c,d\right)=1/512\left[\begin{array}{cccc} 440 & 165 & 165 & 72\\ -192 & 440 & -72 & 192\\ -192 & -72 & 440 & 192\\ 72 & -165 & -165 & 440 \end{array}\right]•\left[\begin{array}{c} a\\ b\\ c\\ d \end{array}\right]$$

(6) $$T_{S}\left(a,b\right)=1/4096\left[\begin{array}{cc} 2821 & 27\\ -27 & 5947 \end{array}\right]•\left[\begin{array}{c} a\\ b \end{array}\right]$$

The expanded results as shown in Equations (7) and (8) were obtained.

(7) $$\begin{array}{ll} f_{POT4\times 4} & \left(\left[\begin{array}{cccc} a & b & c & d\\ e & f & g & h\\ i & j & k & l\\ m & n & o & p \end{array}\right]\right)\\ & =\left[\begin{array}{cccc} \vec{C_{A}}•\vec{V} & \vec{C_{B}}•\vec{V} & \vec{C_{C}}•\vec{V} & \vec{C_{D}}•\vec{V}\\ \vec{C_{E}}•\vec{V} & \vec{C_{F}}•\vec{V} & \vec{C_{G}}•\vec{V} & \vec{C_{H}}•\vec{V}\\ \vec{C_{I}}•\vec{V} & \vec{C_{J}}•\vec{V} & \vec{C_{K}}•\vec{V} & \vec{C_{L}}•\vec{V}\\ \vec{C_{M}}•\vec{V} & \vec{C_{N}}•\vec{V} & \vec{C_{O}}•\vec{V} & \vec{C_{P}}•\vec{V} \end{array}\right] \end{array}$$

Among them:

(8) $$\begin{array}{l} \vec{V}={\left[\begin{array}{cccccccccc} a & b & c & d & e & f\;\cdots \;l & m & n & o & p \end{array}\right]}^{T}\\ \vec{C_{A}}=\left[\begin{array}{ccccc} 0.9843 & -0.2414 & 0.2425\cdots -0.007494 & 0.008556 & -0.01566 \end{array}\right]\\ \vec{C_{B}}=\left[\begin{array}{ccccc} 0.2605 & 0.9218 & -0.08032\cdots -0.2053 & 0.04684 & 0.0105 \end{array}\right]\\ \vec{C_{C}}=\left[\begin{array}{ccccc} -0.2605 & -0.07419 & 0.9214\cdots 0.04638 & -0.1992 & -0.0105 \end{array}\right]\\ \vec{C_{D}}=\left[\begin{array}{ccccc} -0.1367 & 0.2414 & -0.2425\cdots 0.007494 & -0.008556 & -0.1367 \end{array}\right]\\ \vec{C_{E}}=\left[\begin{array}{ccccc} 0.2605 & -0.05081 & 0.05128\cdots 0.05128 & -0.05081 & 0.0105 \end{array}\right]\\ \vec{C_{F}}=\left[\begin{array}{ccccc} 0.05081 & 0.2414 & 0.007494\cdots -0.2425 & -0.008556 & 0.05081 \end{array}\right]\\ \vec{C_{G}}=\left[\begin{array}{ccccc} -0.05081 & 0.008556 & 0.2425\cdots -0.007494 & -0.2414 & -0.05081 \end{array}\right]\\ \vec{C_{H}}=\left[\begin{array}{ccccc} -0.0105 & 0.05081 & -0.05128\cdots -0.05128 & 0.05081 & -0.2605 \end{array}\right]\\ \vec{C_{I}}=\left[\begin{array}{ccccc} -0.2605 & 0.05081 & -0.05128\cdots -0.05128 & 0.05081 & -0.0105 \end{array}\right]\\ \vec{C_{J}}=\left[\begin{array}{ccccc} -0.05081 & -0.2414 & -0.007494\cdots 0.2425 & 0.008556 & -0.05081 \end{array}\right]\\ \vec{C_{K}}=\left[\begin{array}{ccccc} 0.05081 & -0.008556 & -0.2425\cdots 0.007494 & 0.2414 & 0.05081 \end{array}\right]\\ \vec{C_{L}}=\left[\begin{array}{ccccc} 0.0105 & -0.05081 & 0.05128\cdots 0.05128 & -0.05081 & 0.2605 \end{array}\right]\\ \vec{C_{M}}=\left[\begin{array}{ccccc} -0.1367 & -0.008556 & 0.007494\cdots -0.2425 & 0.2414 & -0.1367 \end{array}\right]\\ \vec{C_{N}}=\left[\begin{array}{ccccc} -0.0105 & -0.1992 & 0.04638\cdots 0.9214 & -0.07419 & -0.2605 \end{array}\right]\\ \vec{C_{O}}=\left[\begin{array}{ccccc} 0.0105 & 0.04684 & -0.2053\cdots -0.08032 & 0.9218 & 0.2605 \end{array}\right]\\ \vec{C_{P}}=\left[\begin{array}{ccccc} -0.01566 & 0.008556 & -0.007494\cdots 0.2425 & -0.2414 & 0.9843 \end{array}\right] \end{array}$$

When we substitute a to p all with the same variable *x*, the result is Equation (9):

(9) $$\begin{array}{ll} f_{POT4\times 4} & \left(\left[\begin{array}{cccc} x & x & x & x\\ x & x & x & x\\ x & x & x & x\\ x & x & x & x \end{array}\right]\right)\\ & =\left[\begin{array}{cccc} 0.686^{*}x & 0.684^{*}x & 0.693^{*}x & 0.691^{*}x\\ 0.684^{*}x & 0.678^{*}x & 0.7^{*}x & 0.693^{*}x\\ 0.693^{*}x & 0.7^{*}x & 0.678^{*}x & 0.684^{*}x\\ 0.691^{*}x & 0.693^{*}x & 0.684^{*}x & 0.686^{*}x \end{array}\right] \end{array}$$

Using the same method as Equation (2) and Figure 13b, the inverse 4 × 4 POT can be expanded; the result is Equation (10).

(10) $$\begin{array}{ll} f_{IPOT4\times 4} & \left(\left[\begin{array}{cccc} x & x & x & x\\ x & x & x & x\\ x & x & x & x\\ x & x & x & x \end{array}\right]\right)\\ & =\left[\begin{array}{cccc} 1.458^{*}x & 1.458^{*}x & 1.445^{*}x & 1.445^{*}x\\ 1.458^{*}x & 1.458^{*}x & 1.445^{*}x & 1.445^{*}x\\ 1.445^{*}x & 1.445^{*}x & 1.458^{*}x & 1.458^{*}x\\ 1.445^{*}x & 1.445^{*}x & 1.458^{*}x & 1.458^{*}x \end{array}\right] \end{array}$$

**Figure 14:**
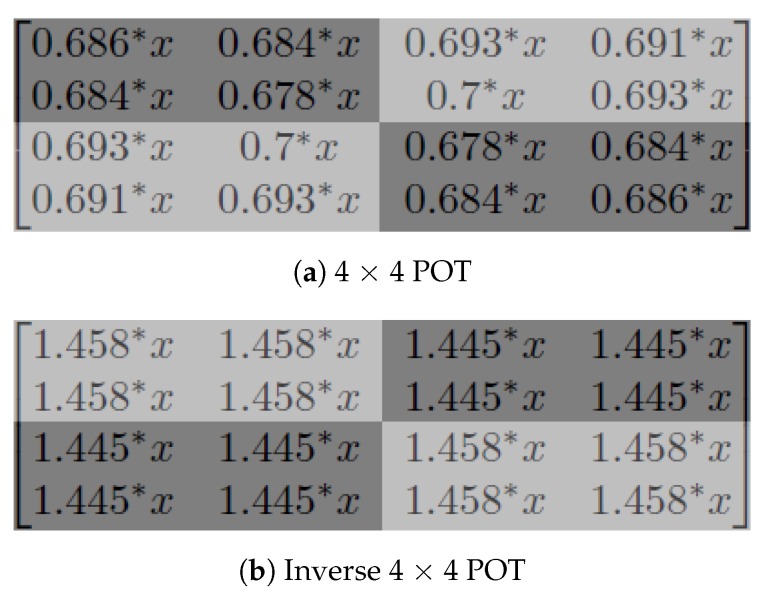
Chequerboard artefact distribution of 4 × 4 POT and inverse 4 × 4 POT.

A look at Equations (9) and (10) shows that both 4 × 4 POT and inverse 4 × 4 POT resulted in a chequerboard-like distribution, as shown in Figure 14. The values are displayed in greyscale. Assuming x>0, in the 4 × 4 POT (Figure 14a), the top left and bottom right corners have lower values (colour is dark) while the top right and bottom left corners have higher values (colour is lighter). The inverse 4 × 4 POT, on the other hand, resulted in exactly the opposite chequerboard colours. The mirrored results appear in Figure 14b.

To further understand how the chequerboard phenomenon occurred, we followed the encoding process described in Figure 4. First, the 4 × 4 PCT (represented as $f_{PCT4\times 4}$) was applied to the 4 × 4 POT result from Equation (9). Note that since application areas of POT and PCT interleave (Section 2.2), the input array was relatively displaced. The operation result is shown in Equation (11).

(11) $$\begin{array}{ll} f_{PCT4\times 4} & \left(\left[\begin{array}{cccc} 0.678^{*}x & 0.684^{*}x & 0.693^{*}x & 0.7^{*}x\\ 0.684^{*}x & 0.686^{*}x & 0.691^{*}x & 0.693^{*}x\\ 0.693^{*}x & 0.691^{*}x & 0.686^{*}x & 0.684^{*}x\\ 0.7^{*}x & 0.693^{*}x & 0.684^{*}x & 0.678^{*}x \end{array}\right]\right)\\ & =\left[\begin{array}{cccc} 2.75^{*}x-0.5 & 0.5 & 0 & 0\\ 0.5 & 0.5 & 0 & 0\\ 0 & 0 & -0.001^{*}x & 0.0004^{*}x\\ 0 & 0 & 0.0004^{*}x & -0.0254^{*}x \end{array}\right] \end{array}$$

We know that PCT and DCT are similar [24]. An image is transformed from the spatial domain to the frequency domain. The distribution of transformed data depends on its frequency. For example, in Equation (11), the top left corner is Low-Pass (LP), while the others are High-Pass (HP). In Equation (11), the value distribution was chequerboard-like due to the previous 4 × 4 POT. After the 4 × 4 PCT, some weak signals were generated in the HP areas. Still, most of the signals were concentrated in the LP area, or the top left of Equation (11) ($2.75^{*}x-0.5$).

**Figure 15:**
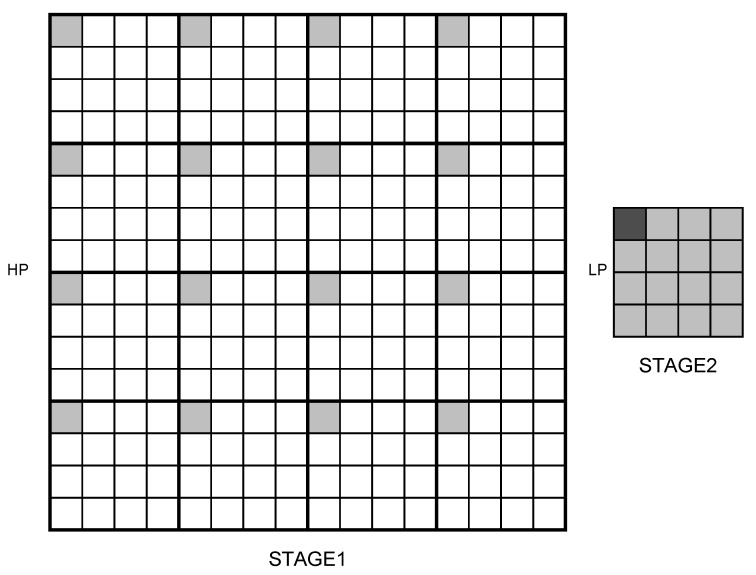
Two-stage PCT in JPEG-XR.

From [24], we know that JPEG-XR has a two-stage PCT, as shown in Figure 15. Each 4 × 4 block processes the first-stage PCT, resulting in an LP coefficient (light-grey) and 15 HP coefficients (white). Then, the second-stage transform was applied to the 16 DC coefficients collected into a single 4 × 4 block. These yielded 16 new coefficients, referred to the LP coefficient of the original block, respectively.

We now apply the second-stage 4 × 4 PCT to a 4 × 4 matrix, filling the LP value of the first-stage transform from Equation (11). The result is shown in Equation (12).

(12) $$\begin{array}{r} f_{PCT4\times 4}(\left[\begin{array}{cccc} 2.75^{*}x-0.5 & 2.75^{*}x-0.5 & 2.75^{*}x-0.5 & 2.75^{*}x-0.5\\ 2.75^{*}x-0.5 & 2.75^{*}x-0.5 & 2.75^{*}x-0.5 & 2.75^{*}x-0.5\\ 2.75^{*}x-0.5 & 2.75^{*}x-0.5 & 2.75^{*}x-0.5 & 2.75^{*}x-0.5\\ 2.75^{*}x-0.5 & 2.75^{*}x-0.5 & 2.75^{*}x-0.5 & 2.75^{*}x-0.5 \end{array}\right])\\ =\left[\begin{array}{cccc} 11^{*}x-2.5 & 0.5 & 0 & 0\\ 0.5 & 0.5 & 0 & 0\\ 0 & 0 & 0 & 0\\ 0 & 0 & 0 & 0 \end{array}\right] \end{array}$$

In Section 2.1, we learned that when QP > 1, values became distorted after the process of quantization and inverse quantization. We set $LP_{S2}$ instead of the LP value in Equation (11), which has been processed with the second-stage 4 × 4 PCT, $C_{0}=11^{*}x-2.5$. We then used QP = 61 to perform quantization and inverse quantization for Equations (11) and (12) (shown as $f_{Q.IQ}\left(\right)$). The results are Equations (13) and (14), respectively. From the source code of DPK [26], we know that under 8-bit greyscale conditions, after undergoing linear adjustments (offset) with the DPK program, the result is |x|≤ 2048. From Figure 3, we can see that when QP = 61 and |c|>144, c∈R, then $f_{Q.IQ}\left(c\right)>0$. This means if we would like $f_{Q.IQ}\left(k^{*}x\right)>0$, k∈R, then |k|≥144/2048, or |k|≥0.0703125. From this, we learn that in the HP area of Equations (11) and (12), the values were adjusted to zero after the $f_{Q.IQ}\left(\right)$ calculation, as they were too small ($<0.0703125^{*}x$). At the end, only the LP portion, or the $f_{Q.IQ}\left(C_{0}\right)$, of the top left corner remained, as shown in Equation (14).

(13) $$\begin{array}{r} f_{Q.IQ}(\left[\begin{array}{cccc} LP_{S2} & 0.5 & 0 & 0\\ 0.5 & 0.5 & 0 & 0\\ 0 & 0 & -0.001^{*}x & 0.0004^{*}x\\ 0 & 0 & 0.0004^{*}x & -0.0254^{*}x \end{array}\right])\\ =\left[\begin{array}{cccc} f_{Q.IQ}\left(LP_{S2}\right) & 0 & 0 & 0\\ 0 & 0 & 0 & 0\\ 0 & 0 & 0 & 0\\ 0 & 0 & 0 & 0 \end{array}\right] \end{array}$$

(14) $$\begin{array}{r} f_{Q.IQ}\left(\left[\begin{array}{cccc} C_{0} & 0.5 & 0 & 0\\ 0.5 & 0.5 & 0 & 0\\ 0 & 0 & 0 & 0\\ 0 & 0 & 0 & 0 \end{array}\right]\right)=\left[\begin{array}{cccc} f_{Q.IQ}\left(C_{0}\right) & 0 & 0 & 0\\ 0 & 0 & 0 & 0\\ 0 & 0 & 0 & 0\\ 0 & 0 & 0 & 0 \end{array}\right] \end{array}$$

Next, we set $C_{1}=f_{Q.IQ}\left(C_{0}\right)$ and performed inverse second-stage PCT 4 × 4 transform on Equation (14) (shown as $f_{IPCT4\times 4}$). The result is Equation (15). One can observe that besides the four corners being $0.25^{*}\left(C_{1}-1\right)$, all other points were equivalent to $0.25^{*}\left(C_{1}+1\right)$.

(15) $$\begin{array}{r} f_{IPCT4\times 4}(\left[\begin{array}{cccc} C_{1} & 0 & 0 & 0\\ 0 & 0 & 0 & 0\\ 0 & 0 & 0 & 0\\ 0 & 0 & 0 & 0 \end{array}\right])\\ =0.25^{*}(\left[\begin{array}{cccc} C_{1}-1 & C_{1}+1 & C_{1}+1 & C_{1}-1\\ C_{1}+1 & C_{1}+1 & C_{1}+1 & C_{1}+1\\ C_{1}+1 & C_{1}+1 & C_{1}+1 & C_{1}+1\\ C_{1}-1 & C_{1}+1 & C_{1}+1 & C_{1}-1 \end{array}\right]) \end{array}$$

From Figure 3, we know that when QP = 61, if $|f_{Q.IQ}\left(c\right)|>0$, then $|f_{Q.IQ}\left(c\right)|\geq 232$, and $C_{1}\;=\;f_{Q.IQ}\left(C_{0}\right)$, or $|C_{1}|\geq 232$; therefore, $C_{1}$ is large enough to make $C_{1}+1\approx C_{1}$ and $C_{1}-1\approx C_{1}$. Then, we can set $LP_{S2}=0.25^{*}C_{1}=C_{2}$ and perform the inverse first-stage PCT 4 × 4 transform on Equation (13). The result is Equation (16):

(16) $$\begin{array}{r} f_{IPCT4\times 4}\left(\left[\begin{array}{cccc} C_{2} & 0 & 0 & 0\\ 0 & 0 & 0 & 0\\ 0 & 0 & 0 & 0\\ 0 & 0 & 0 & 0 \end{array}\right]\right)\\ =0.25^{*}\left(\left[\begin{array}{cccc} C_{2}-1 & C_{2}+1 & C_{2}+1 & C_{2}-1\\ C_{2}+1 & C_{2}+1 & C_{2}+1 & C_{2}+1\\ C_{2}+1 & C_{2}+1 & C_{2}+1 & C_{2}+1\\ C_{2}-1 & C_{2}+1 & C_{2}+1 & C_{2}-1 \end{array}\right]\right) \end{array}$$

Finally, we perform inverse POT on Equation (16) (shown as $f_{IPOT4\times 4}$). The result is Equation (17).

(17) $$\begin{array}{r} f_{IPOT4\times 4}\left(0.25^{*}\left(\left[\begin{array}{cccc} C_{2}-1 & C_{2}+1 & C_{2}+1 & C_{2}-1\\ C_{2}+1 & C_{2}+1 & C_{2}+1 & C_{2}+1\\ C_{2}+1 & C_{2}+1 & C_{2}+1 & C_{2}+1\\ C_{2}-1 & C_{2}+1 & C_{2}+1 & C_{2}-1 \end{array}\right]\right)\right)\\ =\left[\begin{array}{cccc} 0.365^{*}\left(C_{2}-1\right) & 0.365^{*}\left(C_{2}+1\right) & 0.361^{*}\left(C_{2}+1\right) & 0.361^{*}\left(C_{2}-1\right)\\ 0.365^{*}\left(C_{2}+1\right) & 0.365^{*}\left(C_{2}+1\right) & 0.361^{*}\left(C_{2}+1\right) & 0.361^{*}\left(C_{2}+1\right)\\ 0.361^{*}\left(C_{2}+1\right) & 0.361^{*}\left(C_{2}+1\right) & 0.365^{*}\left(C_{2}+1\right) & 0.365^{*}\left(C_{2}+1\right)\\ 0.361^{*}\left(C_{2}-1\right) & 0.361^{*}\left(C_{2}+1\right) & 0.365^{*}\left(C_{2}+1\right) & 0.365^{*}\left(C_{2}-1\right) \end{array}\right] \end{array}$$

**Figure 16:**
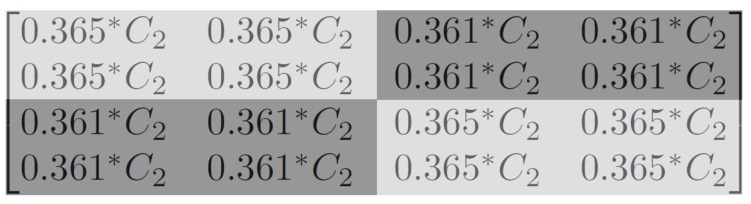
The 4 × 4 region after encoding and decoding; the distribution is chequerboard-like.

The final result of Equation (17) can be rearranged into Figure 16. Assuming $C_{2}>0$ with greyscale representation, the top left and lower right corners have higher values (light grey), while the top right and lower left corners have lower values (dark grey). This distribution is like a chequerboard. Up to now, we have explained the cause for chequerboard artefacts: lossy quantization caused the HP signals to be cleaned out to zero, leaving only the LP signals (Equation (13)). Then, after the inverse 4 × 4 PCT, it can be observed that the results were evenly distributed, or all 16 values were pretty much equal (Equation (16)). At last, the inverse 4 × 4 POT was applied, and a chequerboard-like phenomenon similar to Equation (10) can be seen.

### 2.4. The 4 × 1 POT: Border Artefacts

**Figure 17:**
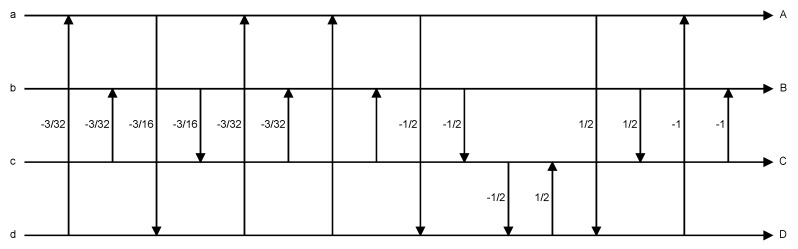
Implementation of 4 × 1 POT.

The implementation of the 4 × 1 POT using the DPK program [26] is shown in Figure 17.

Referring to the method of Equation (1) and Figure 13a, we used a, b, c and d to represent the four points, then substituted them into Figure 17 and expanded. The result is Equation (18)

(18) $$f_{POT4\times 1}\left(\left[\begin{array}{c} a\\ b\\ c\\ d \end{array}\right]\right)=\left[\begin{array}{c} 1.02^{*}a-0.301^{*}b+0.302^{*}c-0.189^{*}d\\ 0.301^{*}a+0.867^{*}b-0.0383^{*}c-0.302^{*}d\\ -0.301^{*}a-0.0369^{*}b+0.867^{*}c+0.302^{*}d\\ -0.188^{*}a+0.301^{*}b-0.302^{*}c+1.02^{*}d \end{array}\right]$$

Similarly, we made a = b = c = d = x, and the result after substitution is Equation (9).

(19) $$f_{POT4\times 1}\left(\left[\begin{array}{c} x\\ x\\ x\\ x \end{array}\right]\right)=\left[\begin{array}{c} 0.8288^{*}x\\ 0.8282^{*}x\\ 0.8303^{*}x\\ 0.8297^{*}x \end{array}\right]$$

Similarly, we can expand the calculation for the inverse 4 × 1 POT where the result is Equation (20).

(20) $$f_{IPOT4\times 1}\left(\left[\begin{array}{c} x\\ x\\ x\\ x \end{array}\right]\right)=\left[\begin{array}{c} 1.207^{*}x\\ 1.207^{*}x\\ 1.205^{*}x\\ 1.205^{*}x \end{array}\right]$$

**Figure 18:**
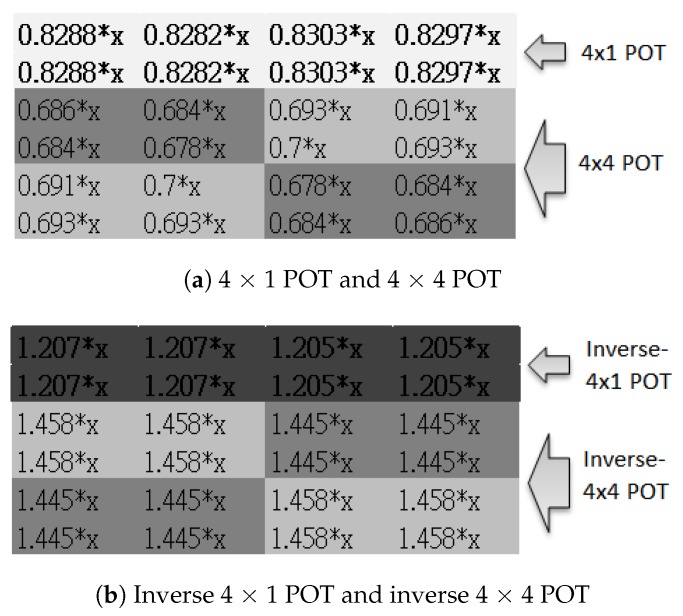
Border artefact distribution of 4 × 1 POT and inverse 4 × 1 POT.

We combined the results of Equations (9), (10), (19) and (20) according to the upper boundary application area of Figure 9. The result is shown in Figure 18. The top two rows are the area where 4 × 1 POT was applied. The bottom four rows are where 4 × 4 POT was applied. One can see that in Figure 18, the value of 4 × 1 POT is bigger than the lower 4 × 4 POT (light grey). In Figure 18b, the value of inverse 4 × 1 POT is less than the lower inverse 4 × 4 POT (dark grey).

Next, let us attempt to understand how border artefacts were formed. Here, we will also consider the interleaving between application areas of POT and PCT. Please refer to Figure 18. To represent the actual state of the application for the upper image boundary, we took two horizontal 4 × 1 POT areas for the top two rows. The next two rows are the top half of the 4 × 4 POT area. This is shown in Equation (21).

(21) $$\left[\begin{array}{cccc} 0.8303^{*}x & 0.8297^{*}x & 0.8288^{*}x & 0.8282^{*}x\\ 0.8303^{*}x & 0.8297^{*}x & 0.8288^{*}x & 0.8282^{*}x\\ 0.693^{*}x & 0.691^{*}x & 0.686^{*}x & 0.684^{*}x\\ 0.7^{*}x & 0.693^{*}x & 0.684^{*}x & 0.678^{*}x \end{array}\right]$$

Using the same steps as Section 2.3, we applied PCT, quantization, inverse PCT and inverse POT operations on Equation (21). The result is Equation (22), where $C_{4\times 1}=f_{Q.IQ}\left(3.04^{*}x-0.5\right)$ and $C_{4\times 4}\;=\;f_{Q.IQ}\left(2.75^{*}x\;-\;0.5\right)$:

(22) $$\left[\begin{array}{cccc} 0.301^{*}C_{4\times 1} & 0.301^{*}C_{4\times 1} & 0.302^{*}C_{4\times 1} & 0.302^{*}C_{4\times 1}\\ 0.301^{*}C_{4\times 1} & 0.301^{*}C_{4\times 1} & 0.302^{*}C_{4\times 1} & 0.302^{*}C_{4\times 1}\\ 0.337^{*}C_{4\times 1} & 0.337^{*}C_{4\times 1} & 0.339^{*}C_{4\times 1} & 0.339^{*}C_{4\times 1}\\ \;\;\;+0.0244^{*}C_{4\times 4} & \;\;\;+0.0244^{*}C_{4\times 4} & \;\;+0.0261^{*}C_{4\times 4} & \;\;\;+0.0261^{*}C_{4\times 4}\\ 0.243^{*}C_{4\times 1} & 0.243^{*}C_{4\times 1} & 0.245^{*}C_{4\times 1} & 0.245^{*}C_{4\times 1}\\ \;\;\;+0.118^{*}C_{4\times 4} & \;\;\;+0.118^{*}C_{4\times 4} & \;\;\;+0.12^{*}C_{4\times 4} & \;\;\;+0.12^{*}C_{4\times 4} \end{array}\right]$$

**Figure 19:**
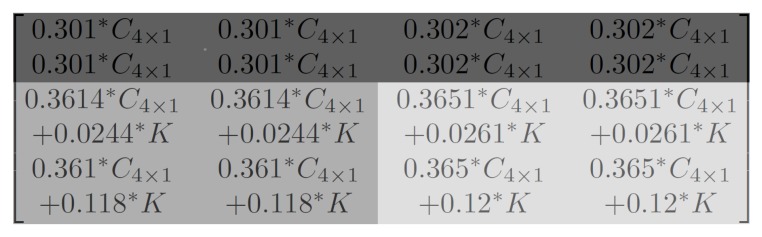
The 4 × 1 region after encoding and decoding, causing border artefacts.

Considering the characteristics of quantization, when x>0, $C_{4\times 1}\geq C_{4\times 4}$. This means we can make $C_{4\times 1}=C_{4\times 4}+K$ where K≥0. Therefore, the result of Equation (22) can be reordered into Figure 19. Assuming $C_{4\times 1}>0$, using greyscale representation, one can see that the top two rows (where 4 × 1 POT was applied) have lower values (dark grey). We only took half (4 × 2) of the bottom two rows (where 4 × 4 POT was applied), so only half of the chequerboard is shown here, but all the values in this area were higher than the top two rows (lighter grey). Up to now, we have explained the cause of border artefacts: this is due to different transformation values resulting from inverse 4 × 1 POT and inverse 4 × 4 POT.

### 2.5. Corner Artefacts

Combining Section 2.3 and Section 2.4, we simulated the top left 4 × 4 area of the image, i.e., not applying POT to the top left 2 × 2 corner, applying 4 × 1 POT to the top right and bottom left corners and applying 4 × 4 POT to the bottom right corner. The result is Equation (23).

(23) $$\left[\begin{array}{cccc} x & x & 0.8288^{*}x & 0.8282^{*}x\\ x & x & 0.8288^{*}x & 0.8282^{*}x\\ 0.8288^{*}x & 0.8288^{*}x & 0.686^{*}x & 0.684^{*}x\\ 0.8282^{*}x & 0.8282^{*}x & 0.684^{*}x & 0.678^{*}x \end{array}\right]$$

We applied the aforementioned 4 × 4 PCT on Equation (23). The result is Equation (24).

(24) $$\begin{array}{r} f_{PCT4\times 4}\left(\left[\begin{array}{cccc} x & x & 0.8288^{*}x & 0.8282^{*}x\\ x & x & 0.8288^{*}x & 0.8282^{*}x\\ 0.8288^{*}x & 0.8288^{*}x & 0.686^{*}x & 0.684^{*}x\\ 0.8282^{*}x & 0.8282^{*}x & 0.684^{*}x & 0.678^{*}x \end{array}\right]\right)\\ =\left[\begin{array}{cccc} 3.34^{*}x-0.5 & 0.5-0.002433^{*}x & 0.2733^{*}x & 0.002163^{*}x\\ 0.5-0.002433^{*}x & 0.5-0.000927^{*}x & 0.0001159^{*}x & -0.1164^{*}x\\ 0.2733^{*}x & 0.0001159^{*}x & 0.005162^{*}x & -0.01103^{*}x\\ 0.002163^{*}x & -0.1164^{*}x & -0.01103^{*}x & 0.0199^{*}x \end{array}\right] \end{array}$$

One can see from Equation (24) that the magnitude of the value change in the top left corner is greater because we did not apply POT to it. Therefore, after undergoing PCT, more signals would be generated in the HP area. Next, we also applied quantization and inverse quantization to Equation (24) (represented by $f_{Q.IQ}\left(\right)$). The result is Equation (25).

(25) $$\begin{array}{rr} f_{Q.IQ} & \left(\left[\begin{array}{cccc} 3.34^{*}x-0.5 & 0.5-0.002433^{*}x & 0.2733^{*}x & 0.002163^{*}x\\ 0.5-0.002433^{*}x & 0.5-0.000927^{*}x & 0.0001159^{*}x & -0.1164^{*}x\\ 0.2733^{*}x & 0.0001159^{*}x & 0.005162^{*}x & -0.01103^{*}x\\ 0.002163^{*}x & -0.1164^{*}x & -0.01103^{*}x & 0.0199^{*}x \end{array}\right]\right)\\ & =\left[\begin{array}{cccc} f_{Q.IQ}\left(3.34^{*}x-0.5\right) & 0 & f_{Q.IQ}\left(0.2733^{*}x\right) & 0\\ 0 & 0 & 0 & f_{Q.IQ}\left(-0.1164^{*}x\right)\\ f_{Q.IQ}\left(0.2733^{*}x\right) & 0 & 0 & 0\\ 0 & 0 & 0 & 0 \end{array}\right] \end{array}$$

Note that the result of Equation (25) is different from Section 2.3 and Section 2.4. The HP signals were not completely transformed to zero. Since $f_{Q.IQ}\left(0.2733^{*}x\right)$ and $f_{Q.IQ}\left(-0.1164^{*}x\right)$ satisfied the aforementioned |k|≥0.0703125, they were kept. The decoding result differed from Section 2.3 and Section 2.4 and is the reason for corner artefacts. Up to now, we have explained the cause of corner artefacts.

## 3. Improvement

**Figure 20:**
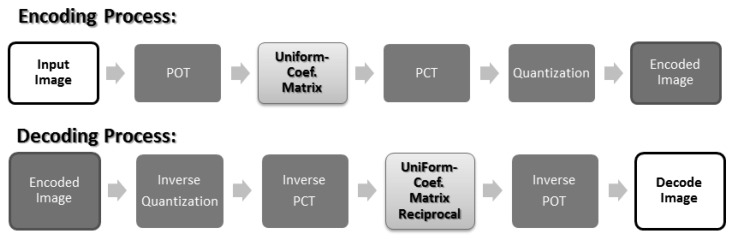
Improved JPEG-XR encoding/decoding process.

From the analysis in Section 2, we discovered three problems in POT: (1) 4 × 4 POT is “uneven” (different values/non-uniform), which causes chequerboard artefacts; (2) the transform result value of 4 × 1 POT is greater than 4 × 4 POT, which causes border artefacts; and (3) POT was not applied on 2 × 2 areas in the four corners, which causes corner artefacts. To solve these problems, we propose the use of a “uniform matrix”. After the POT (encoding) step and before the inverse POT (decoding) step, our uniform matrix was multiplied to even things out. After applying our solution, the process is as described in Figure 20.

### 3.1. Improvement of 4 × 4 POT

As shown in Equation (9), the result of 4 × 4 POT is “uneven”. We hope to obtain a uniform matrix so that the Hadamard product between it and 4 × 4 POT becomes even. We took the average value of 16 points ($0.6887^{*}x$) that have undergone the 4 × 4 POT as our evenness:

(26) $$\begin{array}{r} \left[\begin{array}{cccc} 0.686^{*}x & 0.684^{*}x & 0.693^{*}x & 0.691^{*}x\\ 0.684^{*}x & 0.678^{*}x & 0.7^{*}x & 0.693^{*}x\\ 0.693^{*}x & 0.7^{*}x & 0.678^{*}x & 0.684^{*}x\\ 0.691^{*}x & 0.693^{*}x & 0.684^{*}x & 0.686^{*}x \end{array}\right]⊙\;\left[\mathrm{uniform}\;\mathrm{matrix}\right]\\ =\left[\begin{array}{cccc} 0.6887^{*}x & 0.6887^{*}x & 0.6887^{*}x & 0.6887^{*}x\\ 0.6887^{*}x & 0.6887^{*}x & 0.6887^{*}x & 0.6887^{*}x\\ 0.6887^{*}x & 0.6887^{*}x & 0.6887^{*}x & 0.6887^{*}x\\ 0.6887^{*}x & 0.6887^{*}x & 0.6887^{*}x & 0.6887^{*}x \end{array}\right] \end{array}$$

After calculations, the uniform matrix of 4 × 4 POT is:

(27) $$\left[\begin{array}{cccc} 1.004 & 1.007 & 0.9936 & 0.997\\ 1.007 & 1.016 & 0.9849 & 0.9936\\ 0.9936 & 0.9849 & 1.016 & 1.007\\ 0.997 & 0.9936 & 1.007 & 1.004 \end{array}\right]$$

The corresponding inverse uniform matrix is its reciprocal.

(28) $$\left[\begin{array}{cccc} 1/1.004 & 1/1.007 & 1/0.9936 & 1/0.997\\ 1/1.007 & 1/1.016 & 1/0.9849 & 1/0.9936\\ 1/0.9936 & 1/0.9849 & 1/1.016 & 1/1.007\\ 1/0.997 & 1/0.9936 & 1/1.007 & 1/1.004 \end{array}\right]$$

For the ease of implementation, we adjusted the uniform matrix into a fraction. We set the numerator to 256 ($2^{8}$) and took an approximate value. This way, we can implement using the integer data structure and bit shifting in the program in order to maintain accuracy and decrease complexity. Finally, we get Equations (29) and (30), which are respectively the uniform matrix and inverse uniform matrix of 4 × 4 POT.

(29) $$256/\left[\begin{array}{cccc} 255 & 254 & 258 & 257\\ 254 & 252 & 260 & 258\\ 258 & 260 & 252 & 254\\ 257 & 258 & 254 & 255 \end{array}\right]$$

(30) $$\left[\begin{array}{cccc} 255 & 254 & 258 & 257\\ 254 & 252 & 260 & 258\\ 258 & 260 & 252 & 254\\ 257 & 258 & 254 & 255 \end{array}\right]/256$$

### 3.2. Improvement of 4 × 1 POT

Next, we will attempt to improve the result of 4 × 1 POT. Through [24], we discovered that 4 × 1 POT is a 1D transform, and 4× 4 POT is a 2D transform. This means that 4 × 4 POT has one more dimension than 4× 1 POT, which causes differences in the transformation result. We hope to modify the 4× 1 POT to make its transformation result the same as the 4 × 4 POT. The easiest way is to apply 4× 4 POT instead of 4 × 1 POT on the area, but the problem with this is that a 4× 1 POT area has only four points, while 4 × 4 POT needs 16 points. By observing Equations (7) and (8), we found:

(31) $$\begin{array}{c} \vec{C_{A}}\left[i\right]=\vec{C_{P}}\left[\left(16-i\right)\right]\\ \vec{C_{B}}\left[i\right]=\vec{C_{O}}\left[\left(16-i\right)\right]\\ \vdots \;\;\;\;\;\;\;\;\;\;\;\;\\ \vec{C_{F}}\left[i\right]=\vec{C_{I}}\left[\left(16-i\right)\right]\\ \vec{C_{G}}\left[i\right]=\vec{C_{H}}\left[\left(16-i\right)\right] \end{array}$$

By Equation (31), we proved that 4 × 4 POT is “symmetrical”, as shown in Equation (32). If 4 × 4 POT was performed on an input of 8×2=16 points consisting of eight symmetrical points a–h, the result would also be symmetrical (A–H).

(32) $$f_{POT4\times 4}\left(\left[\begin{array}{cccc} a & b & c & d\\ e & f & g & h\\ h & g & f & e\\ d & c & b & a \end{array}\right]\right)=\left[\begin{array}{cccc} A & B & C & D\\ E & F & G & H\\ H & G & F & E\\ D & C & B & A \end{array}\right]$$

**Figure 21:**
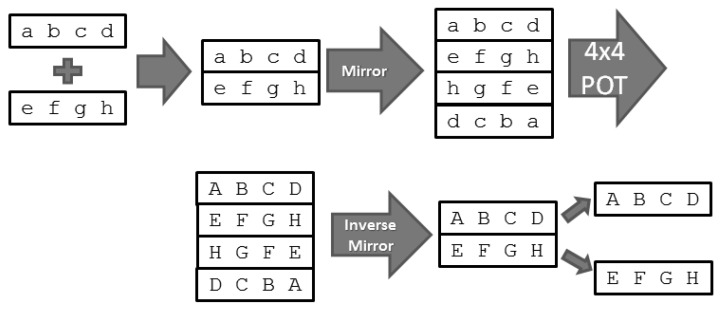
Using 4 × 4 POT instead of 4 × 1 POT.

Due to the symmetrical characteristic of 4 × 4 POT, we can combine the 4 × 1 POT and its neighbouring 4 × 1 POT area to form 4×2=8 points, then expand the area into 4 × 4 POT size through symmetrization (mirroring). Now, the 4 × 4 POT may be applied. The process is as shown in Figure 21. Similarly, we can take advantage of this characteristic when decoding to convert into inverse 4 × 4 POT. To maintain the evenness of the 4 × 4 POT results, it is necessary to multiply the uniform matrix mentioned in Section 3.1 (Equations (29) and (30)) to the process described in Figure 21.

Finally, we have the improvement function $f_{POT4\times 2}$. By making a=b=c=d=e=f=g=h=x, then substituting, this will yield the result described in Equation (33). Its value is $0.6887^{*}x$, which is equivalent to the improved 4 × 4 POT (Equation (26)).

(33) $$\begin{array}{r} f_{POT4\times 2}\left(\left[\begin{array}{cccc} a & b & c & d\\ e & f & g & h \end{array}\right]\right)\\ =\left[\begin{array}{cccc} 0.6887^{*}x & 0.6887^{*}x & 0.6887^{*}x & 0.6887^{*}x\\ 0.6887^{*}x & 0.6887^{*}x & 0.6887^{*}x & 0.6887^{*}x \end{array}\right] \end{array}$$

### 3.3. Improvement of Corner Artefacts (2 × 2 POT)

**Figure 22:**
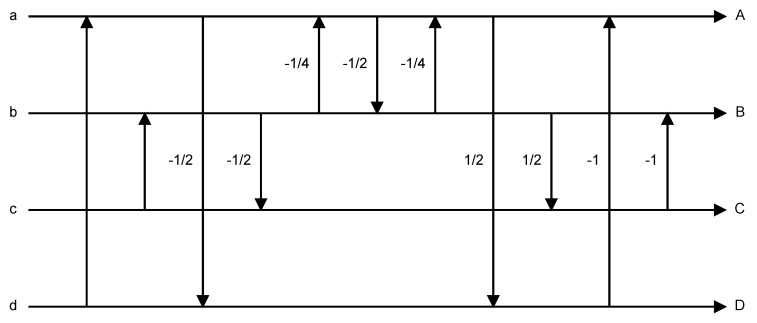
Implementation of 2 × 2 POT.

From the POT application areas marked on Figure 9, we found that there was a 2 × 2 area in each of the four corners where POT was not applied. Their values were different than the areas where POT was applied. This scenario leads to block artefacts in the four corners. In order to ameliorate this phenomenon, we found the implementation of 2 × 2 POT in DPK, as shown in Figure 22.

Here, we also applied the aforementioned expansion method; represent four points with a, b, c and d, substitute into Figure 22 and expand. The result is Equation (34):

(34) $$f_{POT4\times 2}\left(\left[\begin{array}{c} a\\ b\\ c\\ d \end{array}\right]\right)=\left[\begin{array}{c} 1.062^{*}a-0.25^{*}b-0.25^{*}c+0.0625^{*}d\\ -0.2656^{*}a+1.062^{*}b+0.0625^{*}c-0.2656^{*}d\\ -0.2656^{*}a+0.0625^{*}b+1.062^{*}c-0.2656^{*}d\\ 0.0625^{*}a-0.25^{*}b-0.25^{*}c+1.062^{*}d \end{array}\right]$$

We make a=b=c=d=x and substitute to get:

(35) $$f_{POT4\times 2}\left(\left[\begin{array}{c} x\\ x\\ x\\ x \end{array}\right]\right)=\left[\begin{array}{c} 0.625^{*}x\\ 0.5938^{*}x\\ 0.5938^{*}x\\ 0.625^{*}x \end{array}\right]$$

Since we want the same result as that of the aforementioned improved results of 4 × 4 POT and 4 × 1 POT (Section 3.1 and Section 3.2), here the target value was set to $0.6887^{*}x$, i.e.,

(36) $$\begin{array}{r} \left[\begin{array}{c} 0.625^{*}x\\ 0.5938^{*}x\\ 0.5938^{*}x\\ 0.625^{*}x \end{array}\right]⊙\;\left[\mathrm{uniform}\;\mathrm{matrix}\;\mathrm{of}\;\mathrm{POT}\;2\times 2\right]\\ =\left[\begin{array}{c} 0.6887^{*}x\\ 0.6887^{*}x\\ 0.6887^{*}x\\ 0.6887^{*}x \end{array}\right] \end{array}$$

The resulting uniform matrix of POT 2 × 2 is as shown below.

(37) $${\left[\begin{array}{cccc} 1.102 & 1.16 & 1.16 & 1.102 \end{array}\right]}^{T}$$

Inverse uniform matrix:

(38) $${\left[\begin{array}{cccc} 1/1.102 & 1/1.16 & 1/1.16 & 1/1.102 \end{array}\right]}^{T}$$

Finally, we get Equations (39) and (40), which are the uniform matrix and inverse uniform matrix of the 2 × 2 POT in the four missing corners.

(39) $$256/{\left[\begin{array}{cccc} 232 & 221 & 221 & 232 \end{array}\right]}^{T}$$

(40) $${\left[\begin{array}{cccc} 232 & 221 & 221 & 232 \end{array}\right]}^{T}/256$$

## 4. Experimental Results

**Figure 23:**
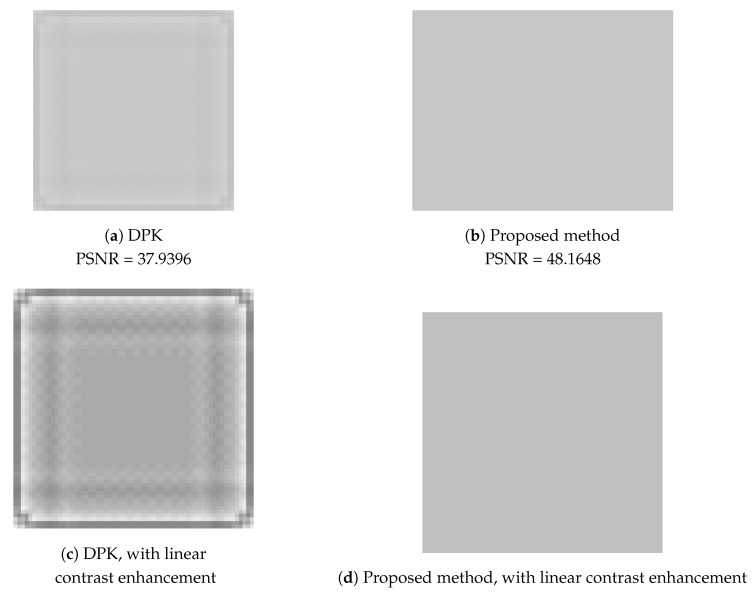
Testing with a monochrome image. After improvement, chequerboard, border and corner artefacts have been eliminated.

First, let us take a look at how a monochrome image has been improved. As shown in Figure 23, one can clearly see that chequerboard, border and corner artefacts have been eliminated.

**Figure 24:**
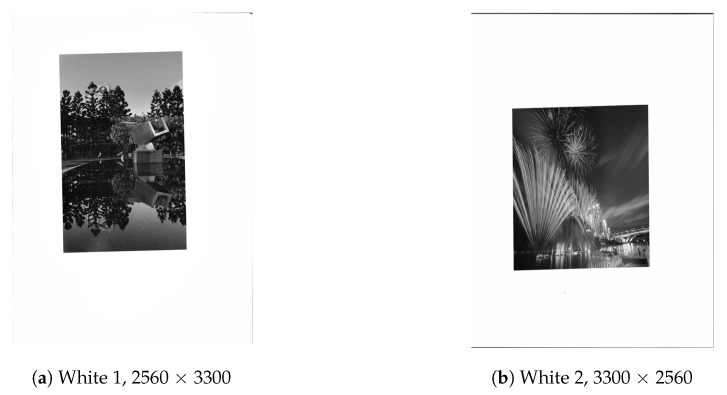
Test images, Part 1.

**Figure 25:**
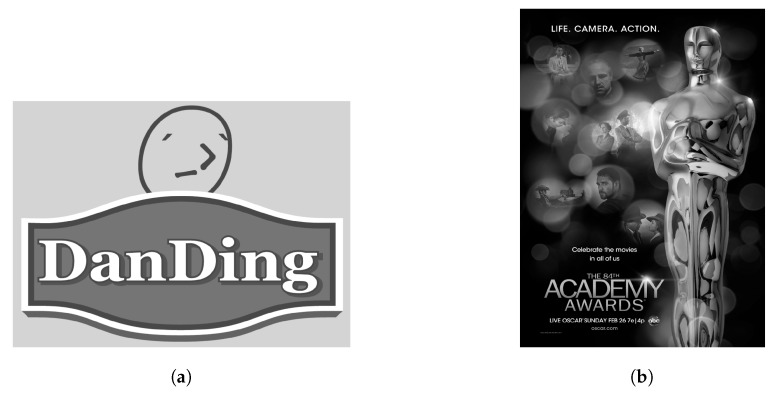
Test images, Part 2. (**a**) logo, 1024 × 768; (**b**) Poster [30], 840 × 1224.

**Figure 26:**
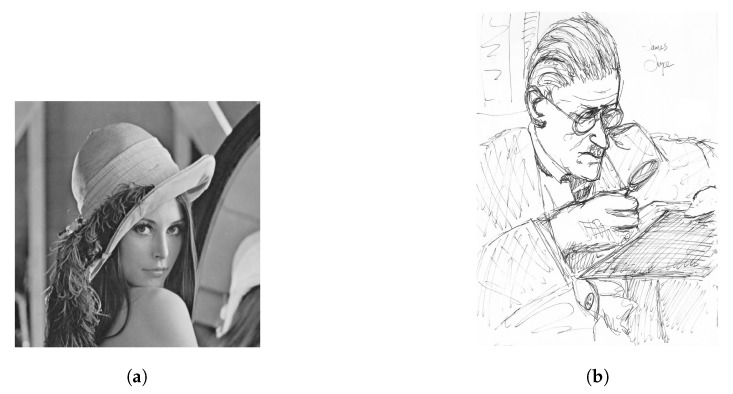
Test images, Part 3. (**a**) Lena, 512 × 512; (**b**) sketch [31], 1080 × 1318.

**Figure 27:**
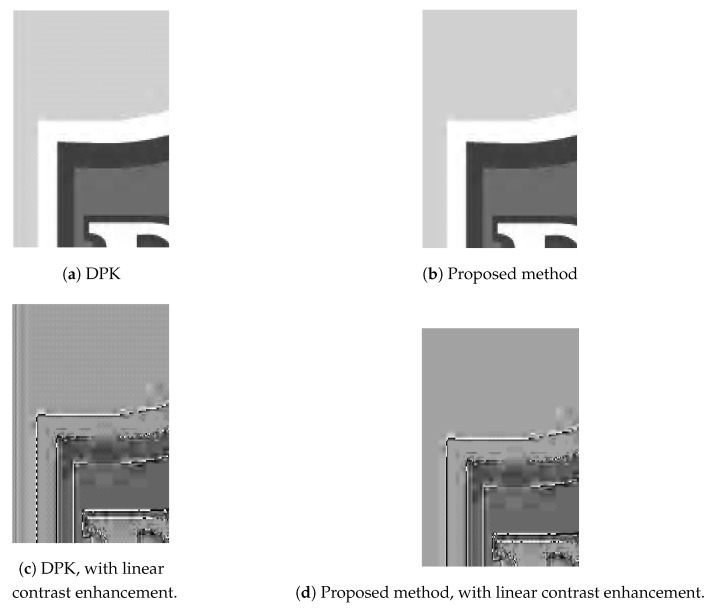
Visual comparison of the logo image (partial, 150 × 150).

**Figure 28:**
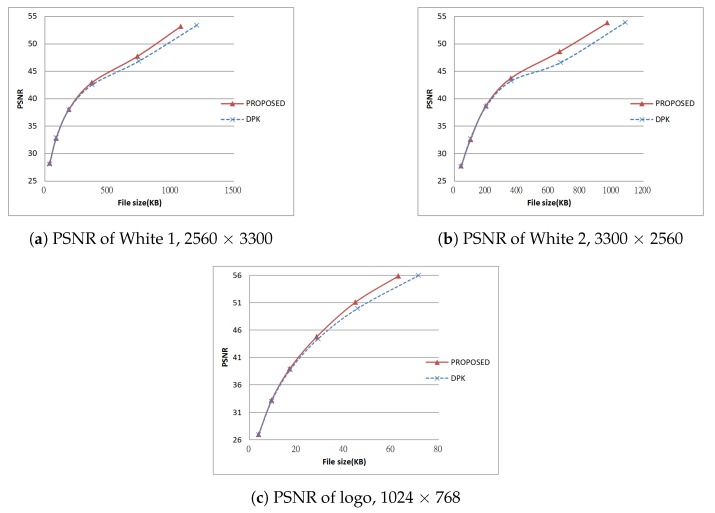
PSNR of test results, Part 1.

**Figure 29:**
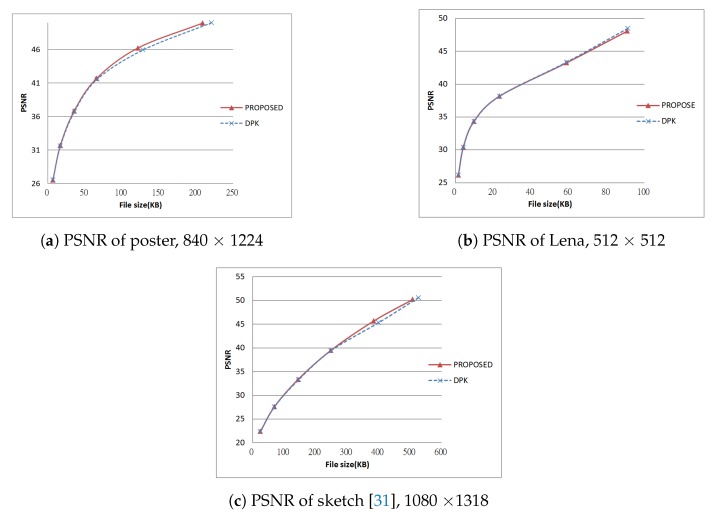
PSNR of test results, Part 2.

**Figure 30:**
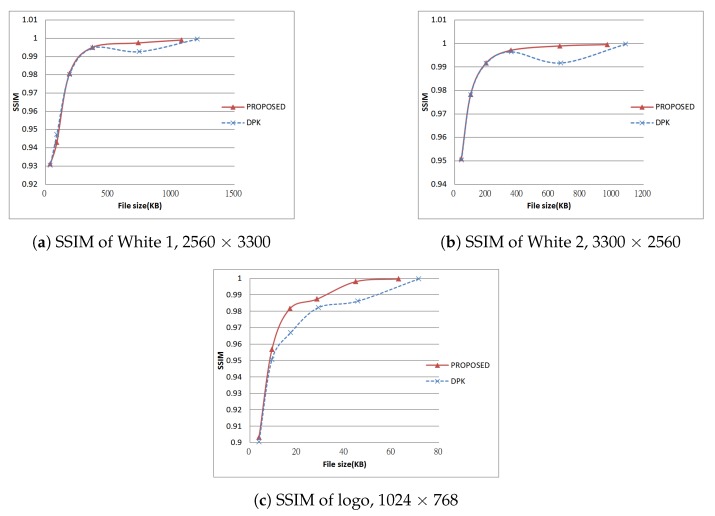
SSIM of test results, Part 1.

**Figure 31:**
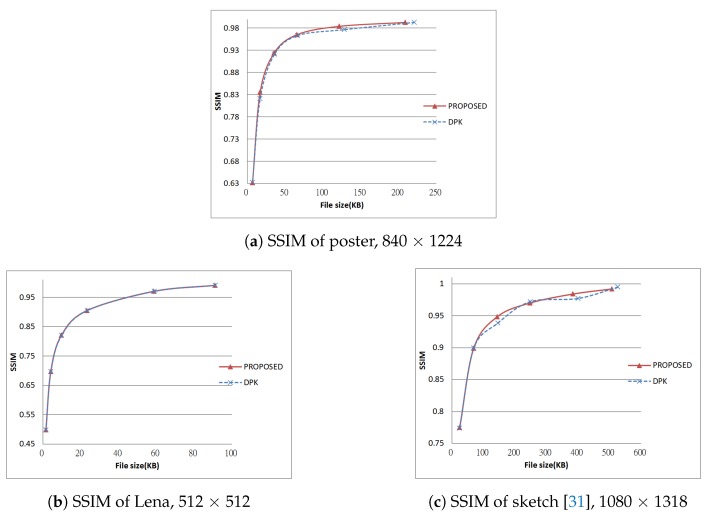
SSIM of test results, Part 2.

Next, we tested using the six images in Figure 24, Figure 25 and Figure 26 (all are 8bpp greyscale images). We performed the JPEG-XR encoding and decoding process using two methods: Microsoft DPK [26] and our improvement program from Section 3. The overlap parameter was fixed at L=1, while the QP was set from 21–101 with an interval of 20. Additionally, 11 were added to observe the performance under a low compression ratio. Calculated separately, PSNR and SSIM [28,29] were used to assess the coding efficiency. The results appear in Figure 27, Figure 28, Figure 29, Figure 30 and Figure 31.

Figure 28a,b and Figure 30a,b are the results of Figure 24 respectively. These two images have wider white borders to simulate photo reproduction and scans. The results show that our improvement method yielded better performance when the compression ratio was medium to low (QP ≤ 41). PSNR increased by as much as 1.97 dB, while SSIM increased by as much as 0.00727. Moreover, when the compression ratio was low (QP ≤ 11), the resulting file size was as much as 10% smaller than DPK.

Figure 28c, Figure 29a, Figure 30c and Figure 31a are the results of Figure 25 respectively. These two images are a common advertising logo and a poster. The results show that PSNR increased by as much as 1.14 dB, while SSIM increased by as much as 0.141. Moreover, when QP = 11, the file size decreased by 5%–10%.

Figure 29b and Figure 31b are the result of Figure 26a using the “Lena” picture (512 × 512 pixel). Using our improvement method, the result was almost the same as the DPK when QP ≥ 50. The reason there was little room for improvement was that chequerboard, border and corner artefacts are insignificant for high-frequency images such as this one.

Figure 29c and Figure 31c are the result of Figure 26b. This is an image of common sketches. in addition, the results show that our improvement method yielded better performance when the compression ratio was medium to low (QP ≤ 40). PSNR increased by as much as 0.34 dB, while SSIM also slightly increased (at most 0.00982).

Figure 27 is the partial visual comparison of the logo image. Figure 27c,d is the result after linear contrast enhancement. One can clearly see that the artefacts appeared in the DPK result (c), and these have been completely eliminated by our proposed method (d).

From these experiments, we learn that under the same compression ratio, our improvement method yielded better results on low-frequency images (mostly white or monochrome, fewer variations) such as Figure 24, Figure 25 and Figure 26. On high-frequency images (less white or monochrome parts, large variations), our performance was about the same as DPK, as seen for the image of Lena. As the chequerboard, border and corner artefacts have been eliminated, the values of neighbouring pixels became more “even”. This effectively increased the compression ratio to produce smaller files.

## 5. Conclusions

JPEG-XR encoding in lossy conditions causes chequerboard, border and corner artefacts. These phenomena are particularly noticeable for higher compression ratios (QP ≥ 41). We discovered that uneven POT results are the cause of these artefacts. We therefore offer a method of improvement, which involves the use of a “uniform matrix”. This method improves the results for 4 × 4, 4 × 1 and 2 × 2 POT, making them “even”. Experiments prove that this method effectively ameliorates chequerboard, border and corner artefacts while yielding the same or better image quality than the original DPK program with no increased calculation complexity or file size. Under the same compression ratio (QP), file sizes may even be smaller. Results show that the proposed improvement method is very effective. 

References
